# Divergent Outcomes of Direct Conspecific Pathogen Strain Interaction and Plant Co-Infection Suggest Consequences for Disease Dynamics

**DOI:** 10.1128/spectrum.04443-22

**Published:** 2023-02-07

**Authors:** Hadjer Bellah, Nicolas F. Seiler, Daniel Croll

**Affiliations:** a Laboratory of Evolutionary Genetics, Institute of Biology, University of Neuchâtel, Neuchâtel, Switzerland; Agroscope

**Keywords:** fungi, competition, virulence, plant pathogens, co-infection, *Zymoseptoria tritici*, wheat

## Abstract

Plant diseases are often caused by co-infections of multiple pathogens with the potential to aggravate disease severity. In genetically diverse pathogen species, co-infections can also be caused by multiple strains of the same species. However, the outcome of such mixed infections by different conspecific genotypes is poorly understood. The interaction among pathogen strains with complex lifestyles outside and inside of the host are likely shaped by diverse traits, including metabolic capacity and the ability to overcome host immune responses. To disentangle competitive outcomes among pathogen strains, we investigated the fungal wheat pathogen Zymoseptoria tritici. The pathogen infects wheat leaves in complex strain assemblies, and highly diverse populations persist between growing seasons. We investigated a set of 14 genetically different strains collected from the same field to assess both competitive outcomes under culture conditions and on the host. Growth kinetics of cocultured strains (~100 pairs) significantly deviated from single strain expectations, indicating competitive exclusion depending on the strain genotype. We found similarly complex outcomes of lesion development on plant leaves following co-infections by the same pairs of strains. While some pairings suppressed overall damage to the host, other combinations exceeded expectations of lesion development based on single strain outcomes. Strain competition outcomes in the absence of the host were poor predictors of outcomes on the host, suggesting that the interaction with the plant immune system adds significant complexity. Intraspecific co-infection dynamics likely make important contributions to disease outcomes in the wild.

**IMPORTANCE** Plants are often attacked by a multitude of pathogens simultaneously, and different species can facilitate or constrain the colonization by others. To what extent simultaneous colonization by different strains of the same species matters, remains unclear. We focused on intra-specific interactions between strains of the major fungal wheat pathogen Zymoseptoria tritici. The pathogen persists in the environment before infecting plant leaves early in the growing season. Leaves are typically colonized by a multitude of strains. Strains cultured in pairs without host were growing differently compared to strains cultured alone. Wheat leaves infected either with single or pairs of strains, we found also highly variable outcomes. Interactions between strains outside of the host were only poorly explaining how strains would interact when on the host, suggesting that pathogen strains engage in complex interactions dependent on the environment. Better understanding within-species interactions will improve our ability to manage crop infections.

## INTRODUCTION

Plants are exposed to a variety of different microbial species acting as pathogens in their natural environment (1–3). Microbial pathogens are often present on hosts in communities with complex interkingdom (mainly bacteria and fungi) interactions, even on individual plants (4–7). Colonization by multiple pathogens is known as a coinfection, and is common in nature (5). Co-infections are of particular interest since the interaction between pathogens can significantly alter infection severity (8). This includes selection for more virulent pathogen strains or species (i.e., causing more severe symptoms), which tend to be more successful in colonizing a host. Because co-infections can also modulate the selection pressure on pathogens, the trajectory of virulence evolution can be modulated by co-infections (9, 10). Co-infecting pathogen species can cause varying levels of damage to the host, depending on the outcome of microbial interactions and host immune responses (11). For example, when plants are co-infected by pathogens of different species, one species tends to suppress the growth and development of the other competing species (12).

The outcome of co-infections can be affected by the order of co-infection (13, 14). Sequential co-infection can decrease the virulence of the second strain involved in the co-infection because of the priming of plant defenses (15), however, such effects can be genotype dependent (14). The host immune defenses activated by the first pathogen might not be mounted rapidly enough to prevent damage (16). However, a secondary infecting pathogen may be limited by primed immune defenses. The secondarily infecting pathogens may benefit though from an overwhelmed host immune system observed e.g., in some viral co-infections (10). Consistent with this phenomenon, simultaneous infections can be more harmful to hosts than sequential infections (17). Interaction among pathogens can also affect the epidemiology of plant diseases, particularly in an agricultural context (18). When multiple pathogens infect a host population, the pathogens can show different transmission dynamics, which in turn can affect the spread of the disease, and the overall impact of the pathogens on the host population. Pathogen cooperation can play a role in co-infections by increasing disease severity, for example, for microbial cooperation increasing disease caused by the ascomycete *Didymella bryoniae* by helping to translocate 4 bacterial species that co-infect Styrian oil pumpkin (19). Hence, interactions between co-infecting pathogens are context-dependent with multiple factors influencing the outcome of infections.

Competition among individuals of the same species can also occur (20) with conflicts over access to nutrients in well-defined niches, such as plant leaves (21). For plants, intraspecific co-infections are playing a key role in disease progression, with fungal-plant pathosystems receiving the most attention (5, 22, 23). Plant pathogenic fungi display a wide range of specialization levels from generalists (able to infect hundreds of host plants) to specialists (which are infectious only on single host species) (24–26). Within pathogen species, strains can show a high degree of variation in virulence (22, 23). The virulence of co-infecting *Podosphaera plantaginis* strains can be increased on the *Plantago lanceolata* host (5). In other systems, such as Zymoseptoria tritici infecting wheat, virulence appears unaffected by co-infections (22, 23); however the trajectory of an infection depends on the host genotype and genotypes of competing strains (22, 27). Besides, the competitive advantage of individual strain genotypes for transmission is not associated with virulence among the strains (27). The growth of mixed genotypes outside of the host is only poorly associated with the success of the same genotype mix on the plant (27). Hence, pathogens with complex life cycles encountering other strains, both prior and during contact with a host plant, may show variable competitive behavior, depending on the environment. Such competitive abilities of the pathogen may have consequences for the plant host, and could impact evolutionary trajectories of host-pathogen systems.

*Z. tritici* is a major fungal pathogen of wheat, causing the economically important foliar disease Septoria tritici blotch (STB) (28). The pathogen constitutes an important component of the microbiome associated with wheat plants (29). *Z. tritici* is thought to overwinter in the soil as spores or mycelium (30). Epidemics develop both through wind-dispersed ascospores originating from sexual reproduction on crop residues, and the splash-dispersal of (asexual) spores during the wheat growing season. On the wheat host, *Z. tritici* exhibits a symptomless phase after coming into contact with wheat leaves lasting 8 to 11 days postinfection, during which the fungus does not induce visible host defense responses (31, 32). In later stages, the fungus invades the intercellular space of leaves producing lesions and asexual fruiting bodies, called pycnidia. Field populations are estimated to include thousands to millions of distinct genotypes (33). The pathogen maintains high genetic and phenotypic diversity worldwide due to the large populations sizes, and significant gene flow among populations as well as frequent sexual reproduction (34–36). Co-infection of wheat leaves by multiple strains of *Z. tritici* are frequent in the field, and leaves are typically colonized by dozens of individual genotypes found at close distance (37). Experiments using 4 strains from the same wheat field showed that co-infection tends to reduce pathogen reproduction (i.e., transmission), most likely due to direct or indirect competition among pathogen strains (22). A subsequent study on the same strain set showed that transmission success of co-infecting strains between hosts was mainly dependent on the ability to produce spores, but not on growth properties on culture medium or damage caused to the host (27). The diversity in co-infection outcomes among a small number of interacting strains raises questions, such as what the dominant co-infection outcomes in more diverse pathogen populations are. Furthermore, questions arise from how strongly the host environment influences the interaction between individual strains.

The aim of this study was to use a set of nearly a hundred pairwise interactions stemming from a representative set of over a dozen field-collected strains to assess the effects of strain co-existence in *Z. tritici.* Given the extensive period of the life cycle spent outside of the host, we first assessed whether growth on culture medium was affected by the direct contact among strains. To assess the effects of co-infection on the wheat host, we analyzed the amount of leaf damage (i.e., lesions) caused by single strain infections versus co-infections. If damage to the plant leaf is primarily a function of the ability of the pathogen to rapidly colonize and grow, we expect to find largely consistent effects of strain interactions on culture medium compared to plant leaves. Increased or decreased damage due to plant infections is expected if the interaction with the plant immune system is modulated by the interaction of co-infecting strains.

## RESULTS

### *In vitro* growth rate assessment and divergent outcomes of cocultures.

We analyzed the growth of 14 *Z. tritici* strains individually (Table S1), and in 91 pairwise combinations (Table S2). A 15th strain used in further experiments had to be excluded due to a contamination issue. The strains originate from an experimental wheat field and are a subset of a larger collection of whole-genome sequenced strains, which were all collected in the same field and year (38, 39). The sampling covered 12 different cultivars with variable susceptibility to the pathogen. A subset of ~20% of all originally analyzed strains were grouped into pairs of clonal genotypes, likely originating from recent asexual reproduction on wheat leaves (39). However, the strains included in this study were all unrelated (i.e., identity-by-descent consistently ~0 among all pairs) (Fig. S1).

**FIG 1:**
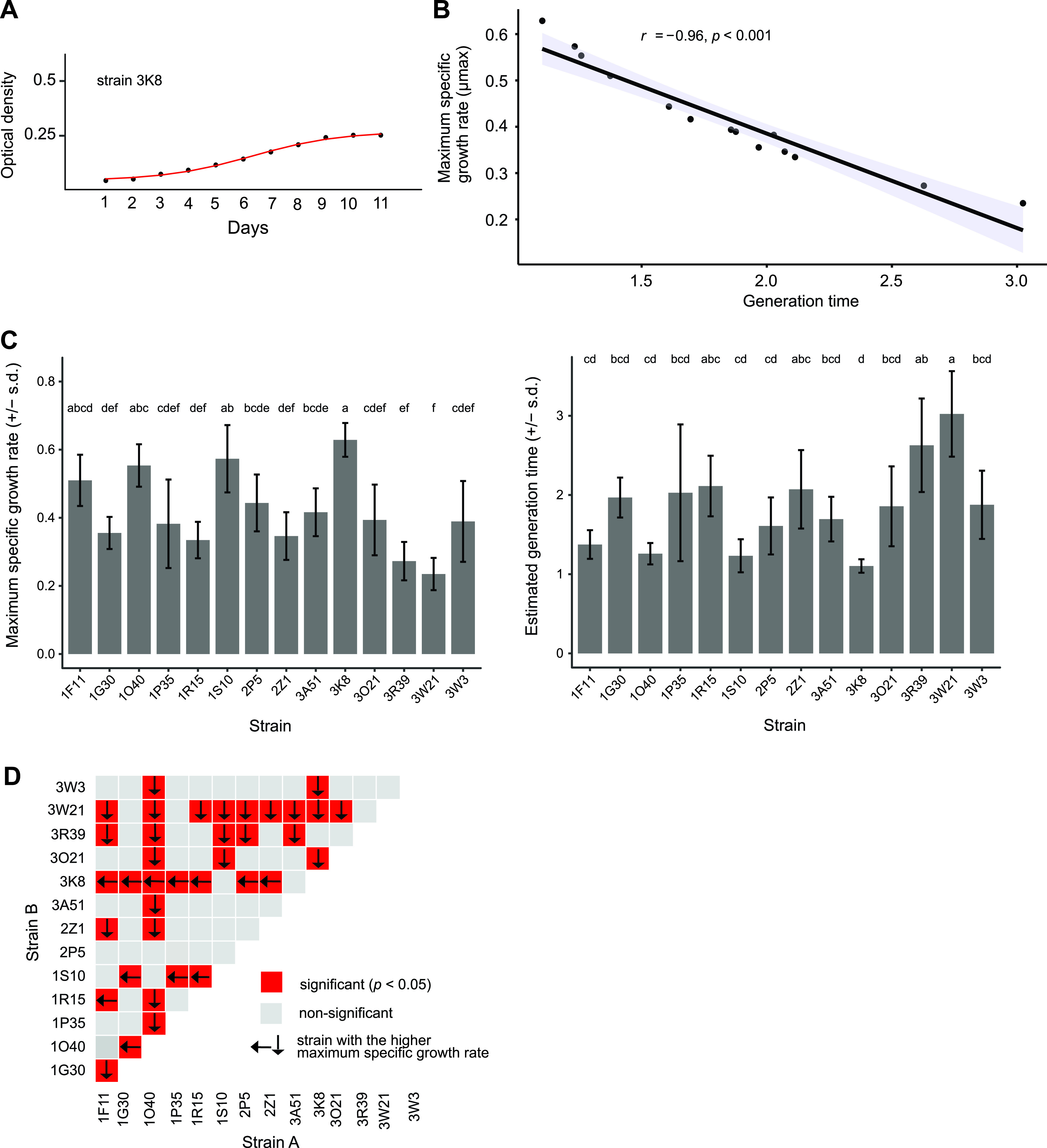
Culture condition growth of Zymoseptoria tritici strains. (A) Growth curve of optical density during the lag, exponential, and stationary phase. A growth plateau was typically reached after ~11 days of incubation in the dark at 18°C. (B) Correlation of growth metrics including the maximum specific growth rate (*μmax*) and doubling time across strains (Pearson correlation, *r* = −0.96, *P-value *< 0.0001). (C) Maximum specific growth rate (*μmax*) and estimated generation time variation among different strains. Significance was assessed using Tukey-Kramer HSD, *P < *0.05. (D) The growth of strains involved in different pairwise interactions assays. Red indicates significant differences between growth rates of individual cultures. The arrow points toward the strain with the faster growth rate.

**FIG 2:**
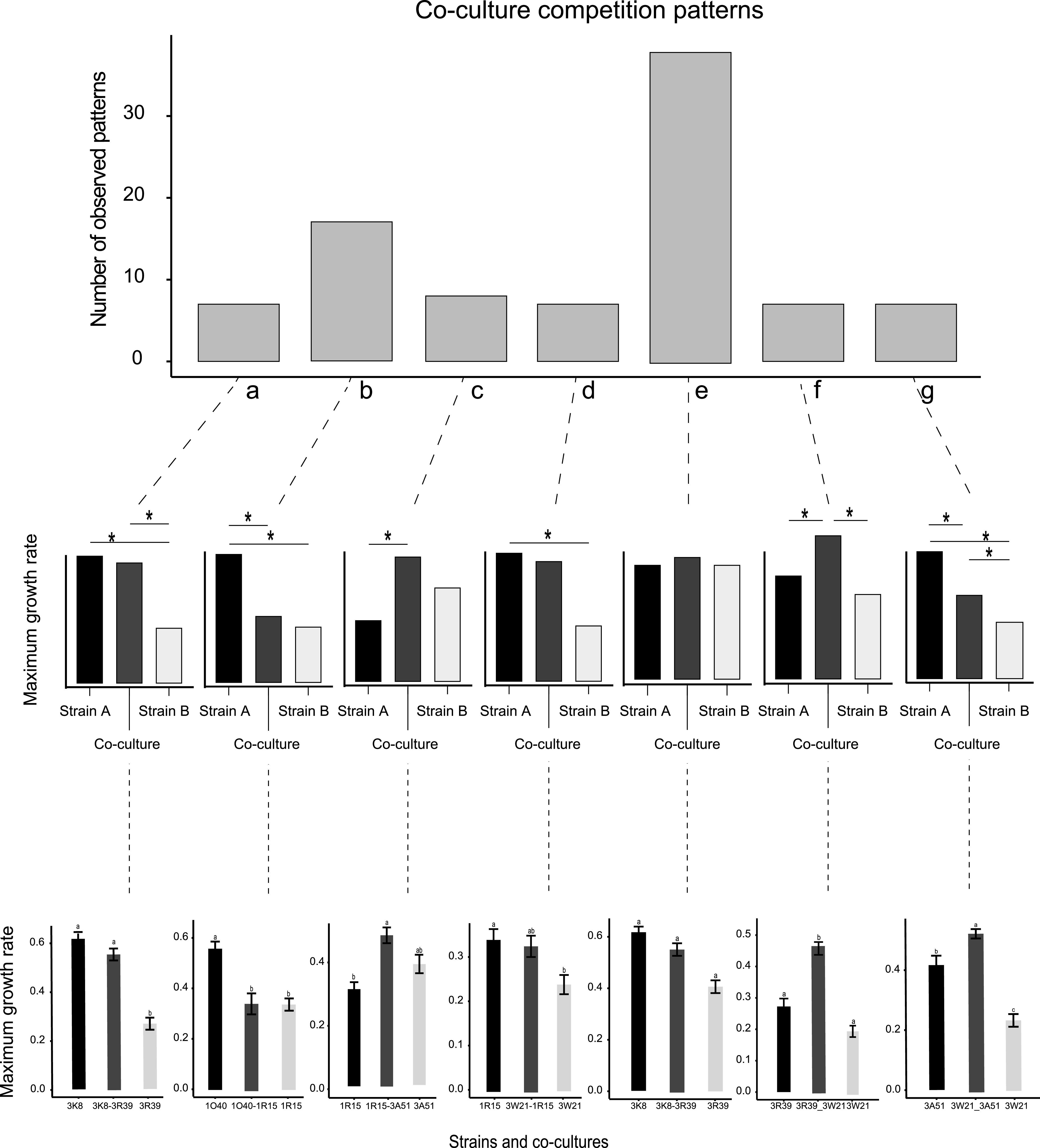
Patterns of single and coculture outcomes observed for the *in vitro* experiments. All interactions were grouped into 7 types of pairwise interaction patterns, depending on whether individual strains showed significant differences to cocultures. Letters (a to g) denote distinct outcomes. Middle row panels show schematics of pairwise interaction patterns indicating for which pairwise comparison a significant difference was observed. Bottom row panels show an example observed in the coculture experiments.

We assessed growth of individual strains in microtiter plates and observed stationary growth after 11 days of incubation at 18°C (Fig. 1A; Fig. S2). We assessed different culture growth metrics, including the maximum specific growth rates (*μmax*), and doubling times. Maximum specific growth rates (*μmax*) and doubling times for different strains were highly correlated (Pearson correlation, *r* = -0.96, *P-value *< 0.0001) (Fig. 1B). Some individual strains displayed significantly different maximum specific growth rates (*μmax*), and estimated generation times compared to other strains (Fig. 1C) (Tukey-Kramer HSD *P < *0.05). We investigated the overall growth in cocultures compared to the growth of individual strains used to establish the coculture. Analyzing the 91 pairs established as cocultures, we found that 37 cocultures showed a significant difference in growth compared to strains grown independently (*P-value *< 0.05) (Fig. 1D). Each of the 14 strains used for the pairings was showing a significantly different growth in at least one pairing (*P-value *< 0.05) (Fig. 1D). The strain 3K8 showed significantly higher maximum specific growth rates (*μmax*) in 9 out of the 13 pairings (*P-value *< 0.05) (Fig. 1D). Strain 1S10 was growing significantly faster in 6 out of 13 pairings (*P-value *< 0.05) (Fig. 1D). In contrast, the strains 1G30, 1P35, 3R39, 3W21, and 3W3 were significantly slower growing compared to all their respective pairings (*P-value *< 0.05) (Fig. 1D). We found that 7 out of 91 cocultures were not growing significantly different to 1 of the 2 strains to establish a mixture, while the other strain grew significantly less alone (Fig. 2A, pattern a, and Fig. S3 and Table S3). Furthermore, 17 out of 91 pairings showed the opposite pattern, with 1 of the strains in the pairing growing significantly more alone compared to the mixture (Fig. 2A, pattern b, and Fig. S3). Additional patterns included either one or both strains growing alone significantly less than the coculture (Fig. 2A, patterns c and f, and Fig. S3). We also observed 7 cocultures growing with no significant differences to both single strain cultures (Fig. 2A, pattern d, and Fig. S3). Besides, we observed 7 cocultures growing at an intermediate level with significant differences to both single strain cultures (Fig. 2A, pattern g, and Fig. S3). Finally, we observed no significant difference in growth among either single strains or the coculture for 38 out of 91 interactions (Fig. 2A, pattern e, and Fig. S3).

### Variation in outcomes among single and coinfections of wheat.

**FIG 3:**
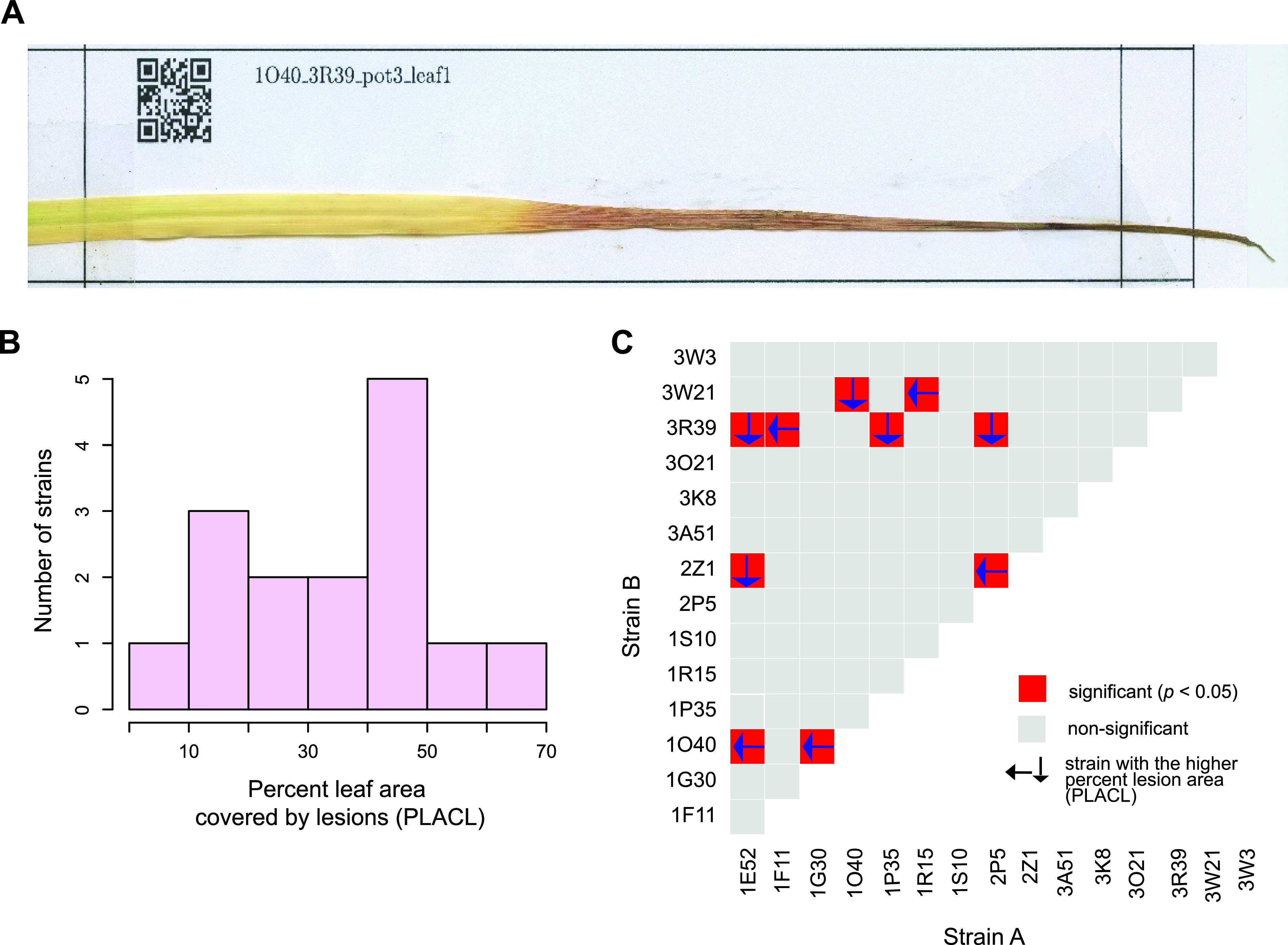
Infection outcomes of single and co-infections of wheat leaves. (A) Scan of a mounted wheat leaf at the end of the infection period. The extent of the lesion area over the entire leaf was assessed. (B) Distribution of lesion area produced by each strain in a single infection experiment. (C) Assessment of co-infection outcomes of all pairs. For significant differences among coculture and single infections, the strain producing the larger lesion area, when alone, is highlighted by an arrow.

**FIG 4:**
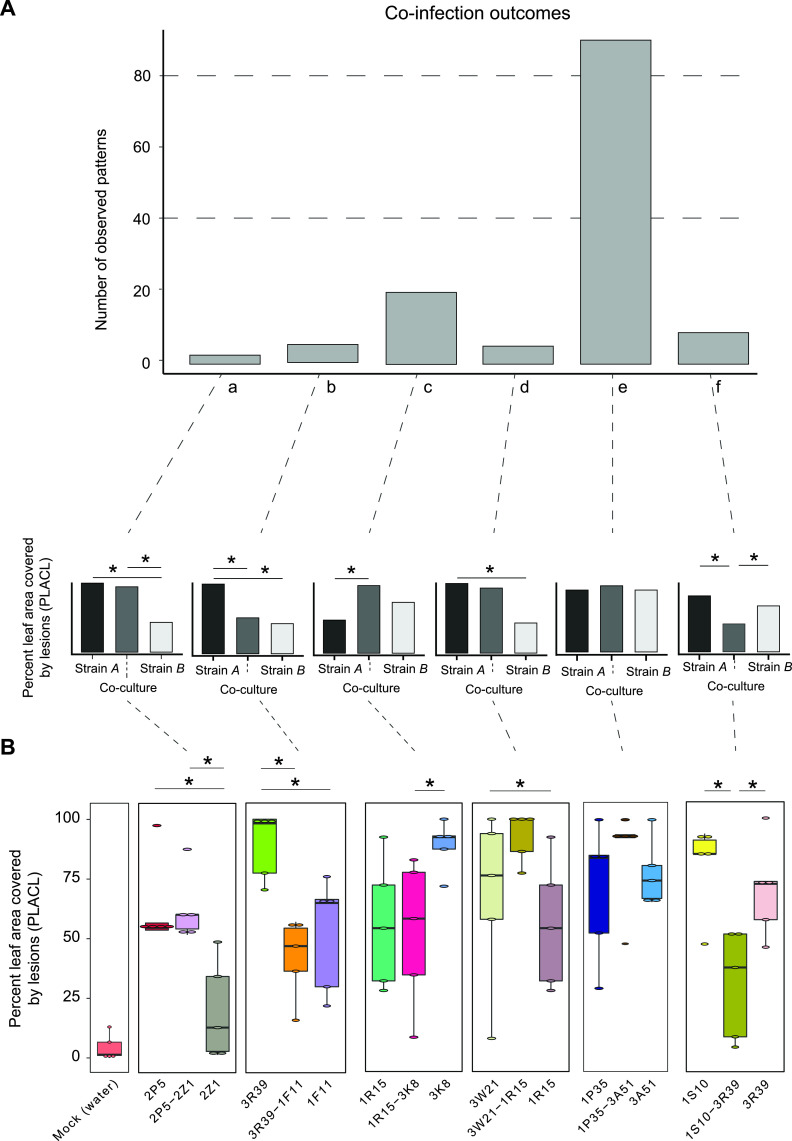
Divergent patterns of co-infection outcomes on wheat leaves. (A) Frequency of different pairwise interactions outcomes grouped into 6 different patterns (a to f). The schematics represent each main pattern. (B) Examples of pairwise interactions of wheat single and co-infection. Water: water controls representing mock inoculations of wheat plants not containing fungal spores. Significance was assessed using Tukey-Kramer HSD, *P < *0.05.

We analyzed infection symptoms on wheat leaves produced both by single strain infections (*n *= 15) and 105 pairs created from the same set of strains. Disease symptoms were assessed as the percent leaf area covered by lesions (PLACL). The measurements were made using scans of entire wheat leaves and contrast-based image analysis (Fig. 3A and Table S4). We analyzed infection outcomes on 5 plants (i.e., biological replicates) for each single or co-infection, and on the first 2 leaves per plant (i.e., technical replicates). Single strain infections produced variable disease symptoms from PLACL ranging from 0 to 70% (Fig. 3B). For the 105 different pairs, 10 pairs included strains with a significant difference in PLACL when infected alone (*P-value *< 0.05) (Fig. 3C). The 10 pairs included pairings of a total of 10 different strains. We analyzed the outcomes of the co-infection compared to the single infections similarly to the culture conditions. We observed 2 and 4 out of 105 pairs with 1 strain being either significantly lower or higher, respectively, compared to the other strain and the co-infection (Fig. 4A and B, patterns a and b). We observed higher numbers of interactions, where either 1 strain alone was producing significantly less symptoms compared to the co-infection, or compared to the other strain (Fig. 4A and B, patterns c and d). Over two thirds (72 out of 105) of all interaction tests showed no significant differences between either strain alone or the co-infection (Fig. 4A and B, pattern e). Finally, the last relevant virulence pattern consisted of 2 strains, which were not statistically different, but the co-infection was significantly different from the 2 strains individually infecting the plant (*P-value < *0.05) (Fig. 4A and B, pattern f). We found that 7 out of 105 combinations followed this pattern. Interestingly, in all tested combinations following this latter pattern, the virulence of co-infection was always lower than observed during single infections.

### Contrasting coculture effects *in vitro* and during host infections.

**FIG 5:**
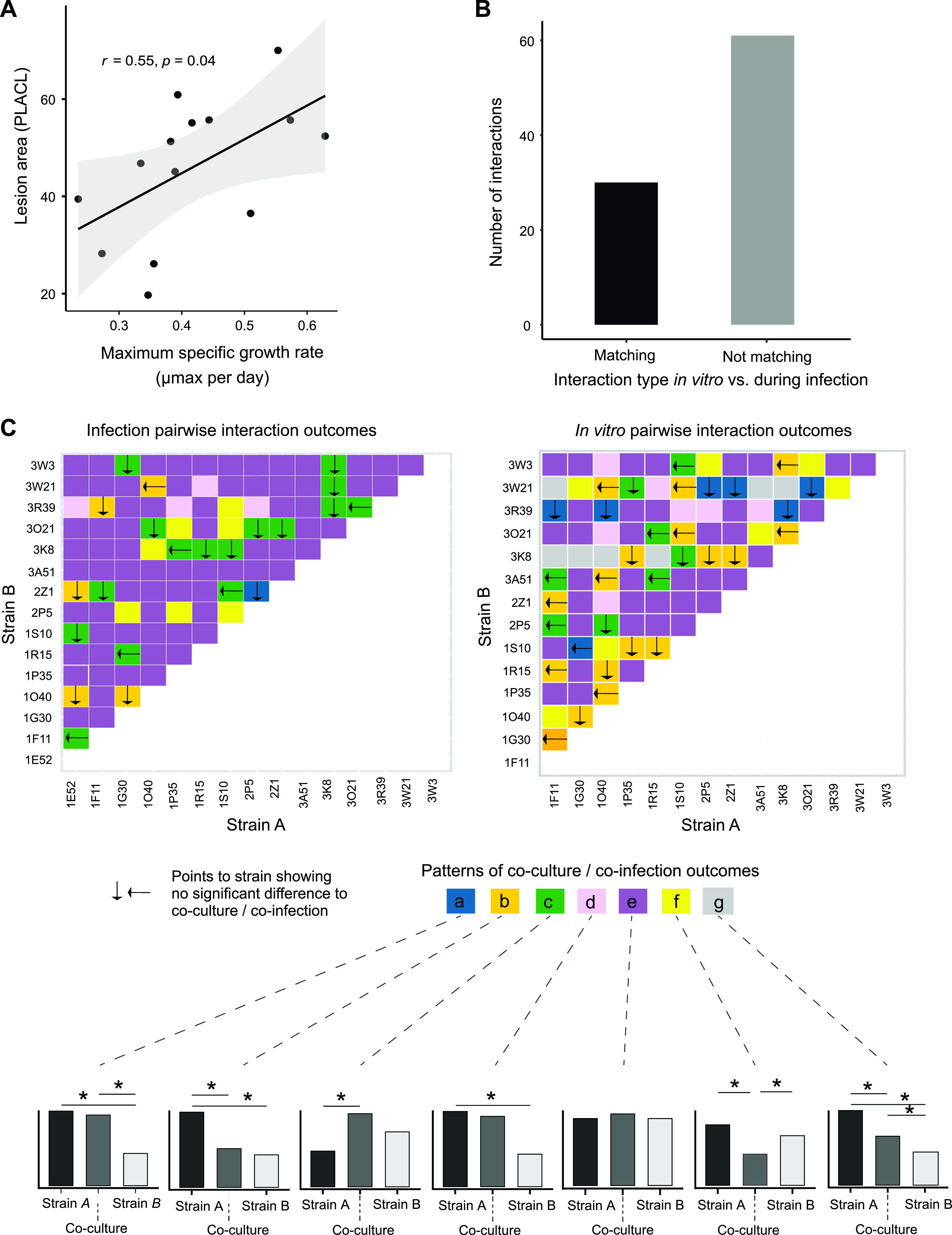
Comparison of *in vitro* versus plant infection pairwise interaction outcomes. (A) Correlation between growth rates of individual strains in culture and lesion development on the plant host. (B) Number of matching and mis-matching pairwise interaction outcomes outside, and on the plant host. (C) The 2 heatmap matrices show plant co-infection and *in vitro* interaction outcomes for each pair of strains. The colors identify specific outcomes whether significant differences were observed in cocultures versus single infections or cultures. The different outcomes were labeled (a to g). The bottom row of panels shows schematic representations of significant growth differences between in cocultures versus single infections or cultures. “Strain A” and “strain B” refer to single infections or cultures, as defined in the heatmaps above.

Strains able to grow rapidly on culture medium may also be able to cause disease symptoms more rapidly on wheat leaves. We found that single strains showed a moderate but significant correlation between maximum growth rate in culture and lesion area development on wheat leaves (*r *= 0.55, *P = *0.04; Fig. 5A). Next, we asked whether patterns of facilitation or inhibition among strains translated from culture medium to wheat leaves. We evaluated the similarity in co-infection versus single infection outcomes of matching strain pairings for a total of 91 strain pairings. We found that, among all evaluated pairs, 30 were found to produce matching interaction patterns, and 61 were not matching between the environments (Fig. 5B, and Fig. S4 and 5). As an example, the strain 3K8 was among the fastest growing strains in single culture; however, 3K8 was not always producing high amounts of lesions on leaves. The strain mixture of 2P5 with 2Z1, in the *in vitro* assay, showed no growth difference, while the infection assay showed mostly more lesion development by 2Z1 compared to 2P5 (Fig. 5C). The interaction between strains 1O40 and 1G30 was similar across conditions, with 1O40 being a fast grower *in vitro* and producing high degrees of lesions in the single strain infection assay. The mixture of 1O40 with 1G30 led to both weak growth in culture, and fewer lesions on leaves (Fig. 5C). Finally, we found the most mixture assays with divergent outcomes between *in vitro* coculture and plant co-infection (Fig. 5C). The mixture 1O40-1F11 produced higher growth than any individual strain, though no difference in lesion development was observed among strains and co-infection. In contrast, the mixture 2P5-1G30 showed no significant difference among any single or mixed culture. The same mixture produced more lesions on leaves than any single strain. Overall, we found that effects of coculture on growth rates outside of the host rarely match predicted effects on lesion development. In particular, co-infection resulting in reduced leaf damage (Fig. 5C, pattern f) was not found in any of the 7 observed cases, which is predicted to be by synergistic or antagnostic growth on culture medium.

## DISCUSSION

Intraspecific co-infections are frequent in nature with plant hosts often being colonized by diverse strain sets (5, 22, 40). Yet, whether co-infections tend to increase or decrease damage to plants is often only known for combinations of a small sets of strains or species. Our study shows that conspecific genotype pairings can produce divergent outcomes whether the interaction is assessed in direct interaction without the host or on the host in a co-infection experiment. Moreover, strains causing more damage to the host are not necessarily showing stronger growth when cultured alone. The potential for different competitive outcomes among strains prior and during host colonization could create complex selection regimes over pathogen life cycles.

We tested for effects of competition by assessing growth of strains grown alone, and combinations thereof on culture medium. Competition can be defined as the interaction occurring when organisms access a limited resource, and having an impact on fitness (41). We found that individual strains showed heritable differences in their ability to grow on nutrient rich medium, which is consistent with previous studies on *Z. tritici* populations (42, 43). Hence, the basic prediction for non-interacting strains in coculture would be a joint growth pattern intermediate to the growth observed for individual strains. This is because we standardized the amount of inoculum across all experiments. A large majority of cocultures produced growth patterns consistent with neutral interactions (i.e., no competition). These cases included outcomes where neither the single nor the coculture showed significant growth differences (Fig. 2, pattern e). Additional cases consistent with this scenario were patterns where the coculture was showing an intermediate growth with either significant or non-significant differences to each monoculture (Fig. 2, patterns a, b, d, and g). For strain pairs showing significant differences in single culture growth, the coculture was often growing similarly to the weaker strain. However, our experiments lacked the statistical power to clearly distinguish growth differences for most pairings. Despite the large number of tested pairs, we observed no coculture with significantly reduced growth compared to both monocultures. Hence, strongly suppressive effects among strains appear to be rare or absent, at least under the tested conditions. Interestingly, a small minority of pairings (~5%) (Fig. 2, pattern f) showed enhanced growth in the coculture compared to each monoculture.

In general, whether such growth acceleration may be triggered by sensing the presence of competitors, and how fungi recognize self from non-self at different morphological stages and under different environmental conditions, remains poorly understood. Over the course of fungal life cycles, non-self-recognition and avoidance are important features of strain interactions (44). Given the fact that all analyzed strains were genetically well distinct, most mycelial interactions are expected to be governed by incompatibility reactions. Non-self-recognition is typically controlled by many distinct genes, defined as vegetative incompatibility loci, that prevent mycelial fusions in most encounters, except self-self-interactions. Only during periods of sexual reproduction, does attraction of opposite mating types become important. Well investigated recognition systems includes vegetative incompatibility reactions at the hyphal stage upon non-self-contact in Neurospora crassa and other filamentous fungi (45, 46). Non-self-recognition may be important if strains were to modulate growth investment depending on conspecific competitors. Recognition of different mating types in *Z. tritici* typically occurs on senescent leaves at the end of the growing season under field conditions (47). Triggering mating under laboratory conditions is challenging, and experimental systems (as used in this study) are unlikely to trigger mating interactions among strains (31).

With the life cycle transition of crop pathogens, such as *Z. tritici* from crop stubbles or the soil to the host plant at the beginning of the growing seasons, strains are likely encountering a number of intra- and interspecific competitors co-infecting the same plant organs. Wheat leaves are known to be typically colonized by multiple genotypes of *Z. tritici* (37), raising the question whether co-infection influences the overall damage caused to the plant host. We focused on lesion development after a successful infection by the pathogen. as a proxy for damage caused to the host. The observed range of lesion area per leaf for single strain infections shows that all strains are capable of causing defense responses on the plant host. We observed no meaningful amount of asexual spore structures (i.e., pycnidia) in contrast to previous greenhouse experiments with the same pathogen (39). The lack of pycnidia production is most likely related to insufficient inoculation time, host resistance or environmental conditions not conducive for full infections. Nevertheless, the extent of lesion development on leaves is correlated with the success of the pathogen to colonize the leaf surface and trigger defenses. Under field conditions, lesion and pycnidia development are correlated (48). We found that most strain pairs used for co-infections showed no significant differences in lesion development either as a single infection or co-infection (Fig. 4, pattern e). The weak differentiation in lesion development among strains is at least partially explained by the higher variance observed among replicates. The reproducible assessment of infection symptoms over the course of multiple weeks is challenging, due to the combination of factors that may potentially affect the final lesion development, including heterogeneity in the application of spores onto leaves, and the initial establishment of the pathogen on leaf surfaces (31). We identified a minority of strain pairs producing intermediate lesion development during co-infection compared to the 2 strains in single infections (Fig. 4, patterns a, b, c, and d). This is consistent with co-infections producing no or only weak deviations in infection outcomes compared to single strain expectations. However, we also found a small set of co-infection pairings where lesion development was reduced, showing that co-infections can decrease the damage to the plant even at identical inoculum density (Fig. 4, pattern f). A reduction in overall leaf damage is consistent with plant resistance mechanisms being able to detect the presence of the pathogen more rapidly. An early activation of antifungal defenses may reduce the spread of the pathogen along the leaf surface and reduce overall lesions. An assessment of fungal biomass would be necessary to ascertain whether the time course of fungal development differs among treatments. Previous work on the same pathogen showed that competitive interactions among strains lead to the elimination of individual strains in single, and over serial, passages (22, 27). Lesion development was not associated with a fitness advantage for transmission (27), suggesting that the reduced plant damage in some co-infections observed in our experiments is not necessarily a disadvantage for the pathogen.

Mechanisms of strain interactions in direct contact in culture medium, and on host plants may differ because of the added complexity of interactions with the plant immune system and other factors.

We found that strain pairs producing faster growth compared to single strains in culture only rarely matched the co-infection effects on lesion development. This mismatch is supported more broadly by having approximately two thirds of all tested co-infection pairs show divergent *in vitro* and plant infection outcomes. Hence, competitive outcomes *in vitro* are only weak predictors for strain interactions on the plant. Such a mismatch between *in vitro* and infection outcomes was also previously observed in an investigation of 4 *Z. tritici* strains (22). Interestingly, pycnidia spore production (a proxy for fitness) was generally reduced during co-infection compared to single strain infections (22). This points to a potential cost of competition at the level of reproduction. However, larger numbers of strain pairings need to be analyzed under conditions allowing for pycnidia observations to draw stronger conclusions. Lesion development may only be weakly associated with reproductive output, as lesions are largely an expression of host immune responses, and damage through reduced photosynthesis. It remains to be investigated how increased lesion development is triggered by the presence of more than one genotype. During the infection process, *Z. tritici* produces hyphae that enter the intercellular space of wheat cells to produce pycnidia in the substomatal space (49, 50). Fluorescent microscopy analyses of co-infections showed that both hyphae and pycnidia are densely packed without apparent exclusion zones among genotypes (27). Increased damage to the host during co-infection could be due to an accelerated hyphal growth of individual strains. Alternatively, different strains may produce different variants of effectors and metabolites during infection (51, 52), which could in combination cause greater damage. It remains unknown whether *Z. tritici* strains can secrete toxins as a response to competitors. An important complement to test hypotheses on mechanisms involved in the competition will be to assess frequencies of individual strains in cocultures and co-infection by means of e.g., a strain-specific discriminant qPCR or fluorescent labeling techniques. A recent study of 4 fluorescence-tagged *Z. tritici* strains showed a consistent pattern of fast-growing strains also growing well in coculture (27). However, expanding either labeling or qPCR approaches to larger pair collections, such as in this study, will be challenging.

The high frequency at which pathogen strains may be exposed to conspecific strains during infection cycles under natural conditions is likely imposing selection pressure for stronger competitive abilities. For instance, selection could favor genotypes retaining growth or infection proficiency, despite the presence of competing strains. We identified a small set of strains retaining growth in each of their pairings with other strains, as well as 2 strains producing similar amounts of lesions, even when exposed to other strains. However, genotyping of cocultures and co-infections would be necessary to assess how well individual strains reproduce when exposed to other strains. Investigating strategies of pathogens coping frequently with high density environments, and exposure to conspecific strains should be an important goal of future co-infection studies. Because co-infections are common in nature (11, 53, 54), understanding the evolution of virulence under co-infection is important for disease management and prevention. Mismatches of competitive outcomes between on and outside of the hosts make predicting co-infection damage more challenging. However, systematic screens of pairwise interactions combined with genetic analyses will help build more informed models of pathogen evolution on crops and other plants. An important realization of recent studies is that host specialization may be a function of competitive abilities for pathogen transmission during co-infection (27). Crop resistance against a specific pathogen is typically assessed by using single pathogen genotypes, or a small set of representative genotypes. Such individual host-pathogen resistance assessments may fail to capture co-infection effects on resistance. Assessing how crop resistance fares under realistic co-infection models may help identify more durable resistance.

## MATERIALS AND METHODS

### Pathogen strain selection and relatedness assessment.

We used 14 and 15 genetically distinct strains of *Z. tritici* for culture (*in vitro*) condition experiments and plant infections, respectively (See Table S1). The strain missing in the *in vitro* experiments had to be excluded due to a contamination issue. All strains were collected from the Field Phenotyping Platform (FIP) site of the ETH Zürich, Switzerland (Eschikon, coordinates 47.449°N, 8.682°E) in 2016 from 8 winter wheat cultivars (55). Strains were selected to avoid pairs of clonal genotypes previously described in the population (39). After sampling, spores of each strain were stored in either 50% glycerol or anhydrous silica gel at − 80°C (39). Illumina sequencing data sets are available for all used strains (39).

Whole-genome sequencing data was previously used to identify single nucleotide polymorphisms (SNPs) segregating among the strains isolated from the Eschikon wheat field site (39). Raw data was made available in NCBI SRA BioProject number PRJNA596434. SNPs were identified by mapping short sequencing reads against the *Z. tritici* reference genome IPO323 (39). We used vcftools v. 0.1.16 (56) to select single random SNPs in 1 kb windows to obtain an even SNP coverage across the genome, and to reduce computational load. We calculated identity-by-descent (IBS) for all pairs of strains available from the Eschikon field site, including the pairs used for interaction assays using TASSEL v. 5.0 (57). The density distribution of IBS values was drawn using *geom_density* in the {ggplot2} R package (58).

### *In vitro* co-cultivation of conspecific strains.

*Z. tritici* strains were revived in solid culture media Yeast-Malt-Agar (YMA) added with 50 μg/mL of kanamycin, and incubated for 8 to 10 days in 18°C. A single colony of each strain was selected and inoculated in a 50 mL conical flask containing 20 mL liquid yeast sucrose broth (YSB) medium supplemented with 50 μg/mL of kanamycin. The inoculated flasks were incubated in the dark at 18° C and at 140 to 180 rpm on an orbitary shaker for 8 days. After 8 days of incubation, the cultures were passed through 4 layers of sterile meshed cheesecloth (Oekostar Textile AG) to eliminate hyphal biomass and retain only spores. Spore suspensions were centrifuged for 15 min at 3700 rpm (Vaudaux-Eppendorf AG). After discarding the supernatant, the pellet was washed with sterile water to remove media traces. The process was repeated one more time. Retained spores were grown in 50 mL conical flask, each containing 20 mL of Vogel’s minimal medium supplemented with 50 μg/mL of kanamycin. The flasks were inoculated with spores to reach a spore density of approximately 10^7^ spores per mL. The cultures were then incubated in the dark at 18° C and 140 to 180 rpm for 10 days. For growth rate monitoring experiments, spores were filtered, diluted in fresh minimal media, and the cell density adjusted to 10^5^ spores/mL by counting the cells in a KOVA cell chamber system (Kova International). Strains were then cultured alone or in pairs in 96-well plates, in either 100 μL or 200 μL, respectively. The plates were sealed and incubated at 18°C at 12 rpm on a shaker incubator for 11 days, with optical density measurements (OD_600_) once daily on a SpectraMax i3x plate reader (Molecular Devices) with a shaking period of 5 s prior to the measurement. The OD was exported to Microsoft Excel for further analysis, and generation of the growth curves. At least 5 replicates for each single or paired culture were set up (Table S3).

### Wheat cultivar selection and infection assays.

The wheat (Triticum aestivum *L.*) cultivar Combin was used, due to its susceptibility to *Z. tritici* (59). Five seeds of the cultivar Combin were placed in pots filled with compost soil, and placed in white watering boxes. The pots were set to replicate (*n *= 5) each treatment of single and co-infection. Wheat was cultivated during 21 days in a growth chamber with a photoperiod of 16 h light/8 h dark, a temperature of 18°C, and a relative humidity of at least 60%. Plants were watered when needed, and randomized regularly. Strains of *Z. tritici* used in the infection experiment were revived from stock in solid culture media YMA supplemented with 50 μg/mL of kanamycin, as described above. Strains were cultured at a temperature of 18° for 8 to 10 days. Following this time period, a single colony of each strain was selected and used to inoculate 20 mL of liquid culture media containing YSB supplemented with 50 μg/mL of kanamycin. The incubation was at 18°C on a shaker for 8 to 10 days at 160 rpm. Following this step, strains were filtrated using sterile cheese cloth (Oekostar Textile AG), and placed in a cold room at 4°C. Then, 3 to 5 days before their use in the experiment, the strains were taken from the cold room, and 500 μL of each strain was used to inoculate 20 mL of liquid YSB and placed in a shaker at 140 to 160 rpm and 18°C. The strain cultures were filtered using sterile cheese cloth (Oekostar Textile AG), and centrifuged for 15 min at 3700 rpm (Vaudaux-Eppendorf AG). After discarding the supernatant, the pellet was resuspended in 20 mL of Milli-Q water (Merck Group), and centrifuged again for 15 min at 3700 rpm. This step was repeated once more. The spore concentration of each strain was adjusted to either 10^5^ spores per mL or half the concentration for co-infections using a hemocytometer (Kova International). The strains were diluted in Milli-Q water (Merck Group) to reach the desired concentrations. Finally, 0.1% of Tween 20 (Sigma-Aldrich) was added to facilitate wheat leaf spore adhesion.

Treatments consisted of plants sprayed with water only (control treatment), plants infected with 1 strain at 10^5^ spores per mL (single strain control), or plants co-infected with 2 strains with a concentration of 5 × 10^4^ spores per mL per strain (co-infection treatment). After 21 days of seed sowing, each pot was sprayed separately protected by cardboard to prevent splashing. Sprayers containing either spore suspensions or sterile Milli-Q water (Merck Group) were used. Each pot was placed in white container boxes and sealed with plastic bags and adhesive tape after being watered. The sealing helps maintaining 100% humidity in a room at 18°C for 48 h. After this period, each plastic bag was opened and left in the climate-controlled room for 21 days. The plants were watered when needed, and pots were regularly randomized within each white box. White boxes were also regularly randomized.

### Plant infection assessments.

After 21 days of infection, the first 2 leaves of each plant were cut and pasted on paper sheets containing a QR code for tracking. No leaves were excluded, regardless of the appearance of symptoms. Each sheet was scanned at 1200 dpi using a flatbed scanner (EPSON perfection V55O) capturing the entire leaf (Fig. 3A). Following this step, an automated image analysis was performed using ImageJ (Rasband, W.S., ImageJ, U.S. National Institutes of Health, https://imagej.nih.gov/ij/, 1997-2018). We used a macro that was developed to assess *Z. tritici* disease symptoms (55), and to determine the percentage of leaf area covered by lesions (PLACL), which is a proxy for leaf damage and virulence (60). Automatic assessments were visually checked for consistency. PLACL values were adjusted if color thresholds were not appropriately distinguishing yellowish (i.e., lesion areas) and green (i.e., healthy) areas on some leaves. Overall, less than ~10 pycnidia at a maximum were confidently observed per leaf during the infection period, and most leaves contained no pycnidia. Hence, pycnidia counts were not considered further.

### Data analyses.

All data analyses were carried out using R v4.0.4 software (61). Optical densities were corrected for the blank control (Vogel’s minimal media) by subtracting the mean optical density for each strain. Fungal growth curves were estimated by fitting a log-logistic model to the data using the {Growthcurver} R package (62). Furthermore, {Growthcurver} was used to summarize the following growth characteristics: generation time (t_d_) and the maximal specific growth rate (*μmax*). Visualizations were made using the {ggplot2} R package (58).

Analysis of single versus coculture data was performed using one-way ANOVA and Tukey-Kramer tests for *post hoc* analysis in R. To satisfy normality assumptions, PLACL data was square-root arcsine transformed. Data handling was performed with the R packages {dplyr} (63), {reshape2} (64), and {tidyr} (65). We used a linear model to test the effect of the different treatments on PLACL, followed by a one-way ANOVA with the function “Anova” from the {car} package (66). Assumptions of linear models were visually assessed with the function “autoplot” from the package {multcomp} (67)*. Post hoc* analysis was done using the “emmeans” function from the package {emmeans} (68) with the Tukey-Kramer method for multiple testing correction.

### Data availability.

All generated data is provided in the supplementary material.
