# Supplementary material for: Divergent Outcomes of Direct Conspecific Pathogen Strain Interaction and Plant Co-Infection Suggest Consequences for Disease Dynamics
Source: Microbiol Spectr. 2023 Feb 7;11(2):e04443-22. doi: 10.1128/spectrum.04443-22 (PMC10101009; doi:10.1128/spectrum.04443-22)
Supplement: Supplemental file 1 — Figures S1-S5. Download spectrum.04443-22-s0001.pdf, PDF file, 1.2 MB [file spectrum.04443-22-s0001.pdf]

Supplementary Figures

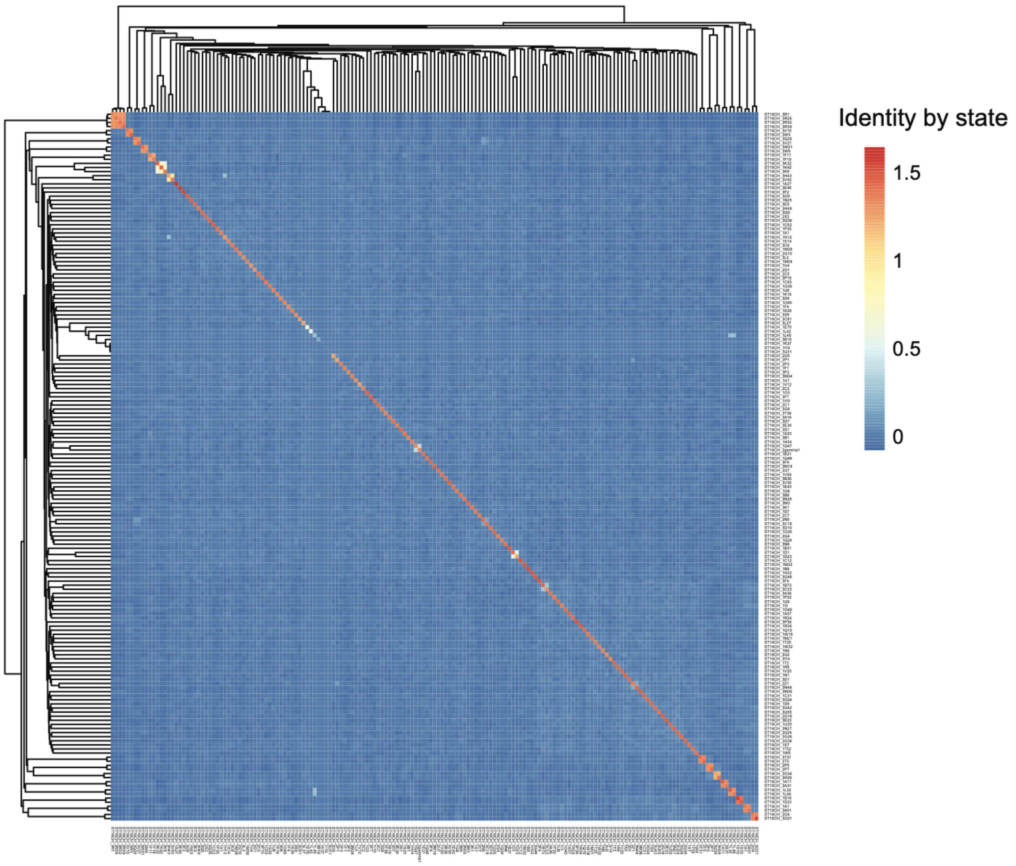

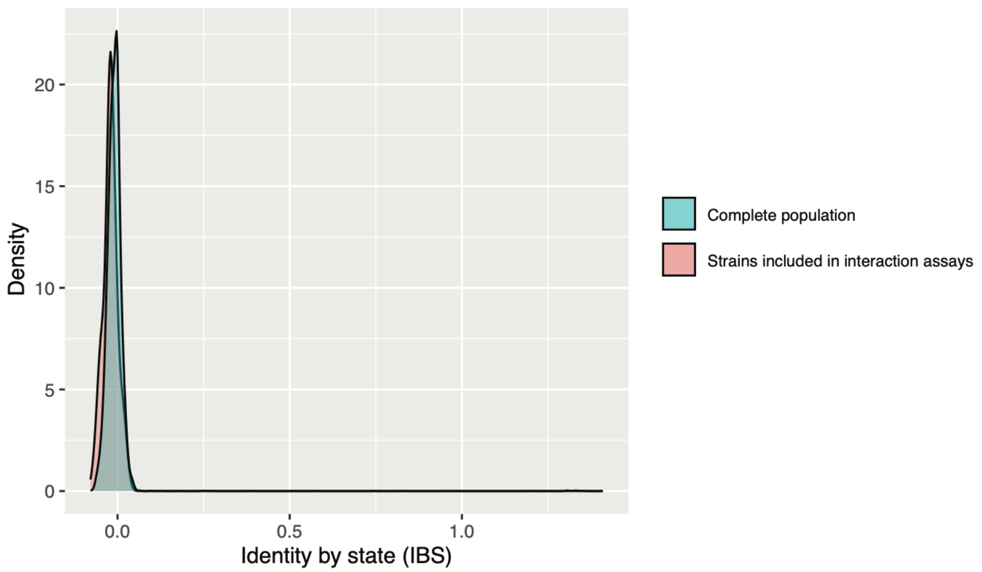

Supplementary Figure S1: Analyses of whole-genome sequencing data of n=173 strains to assess relatedness (expressed as identity-by-state) among pairs of strains. All strains were collected from a single wheat field (Singh et al. 2021). The color scale expresses no relatedness as blue (~0). The diagonal corresponds to self-comparisons and shows maximum relatedness. Strain pairs with near identical genotypes (*i.e.,* clones) are highlighted by orange/red colors of high identity-by-state. The density curves at the bottom contrast the distribution of identity-by-state values for all strain pairs (in green) and strain pairs used for interaction assays (both *in vitro* and on the host). All strain pairs used for interaction assays showed near zero relatedness.

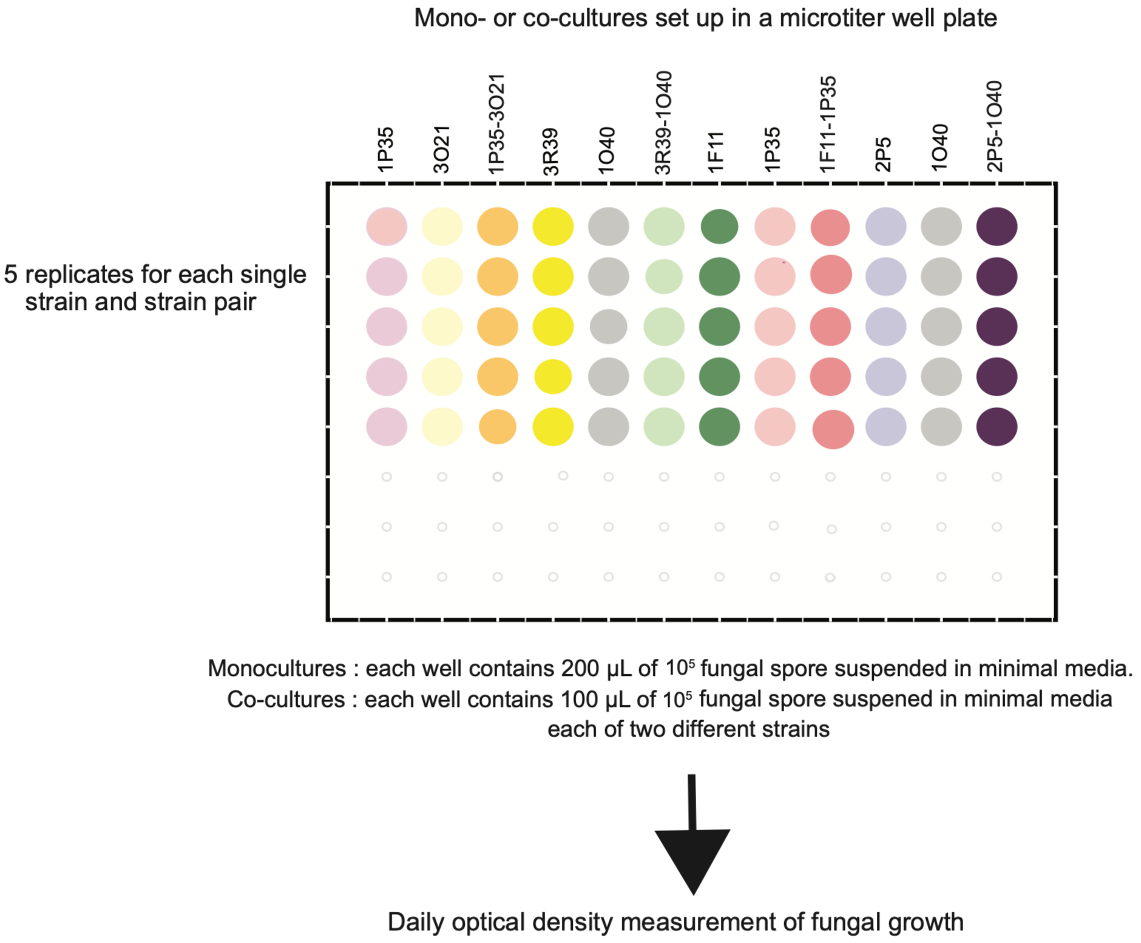

Supplementary Figure S2: Schematic of the *in vitro* culture growth assessment in microtiter plates. A) Schematic overview of *in vitro* fungal strain growth assessment in single and mixed cultures using optical density (OD) measurements. For each monoculture a total volume of 200 µl of fungal spore concentration of 10^5 suspended in minimal media was cultured in each microplate well, whereas for mixed culture, each microplate well contained 100 µl of fungal spores each from two different strains. At least 5 replicates for each single or paired culture were set up.

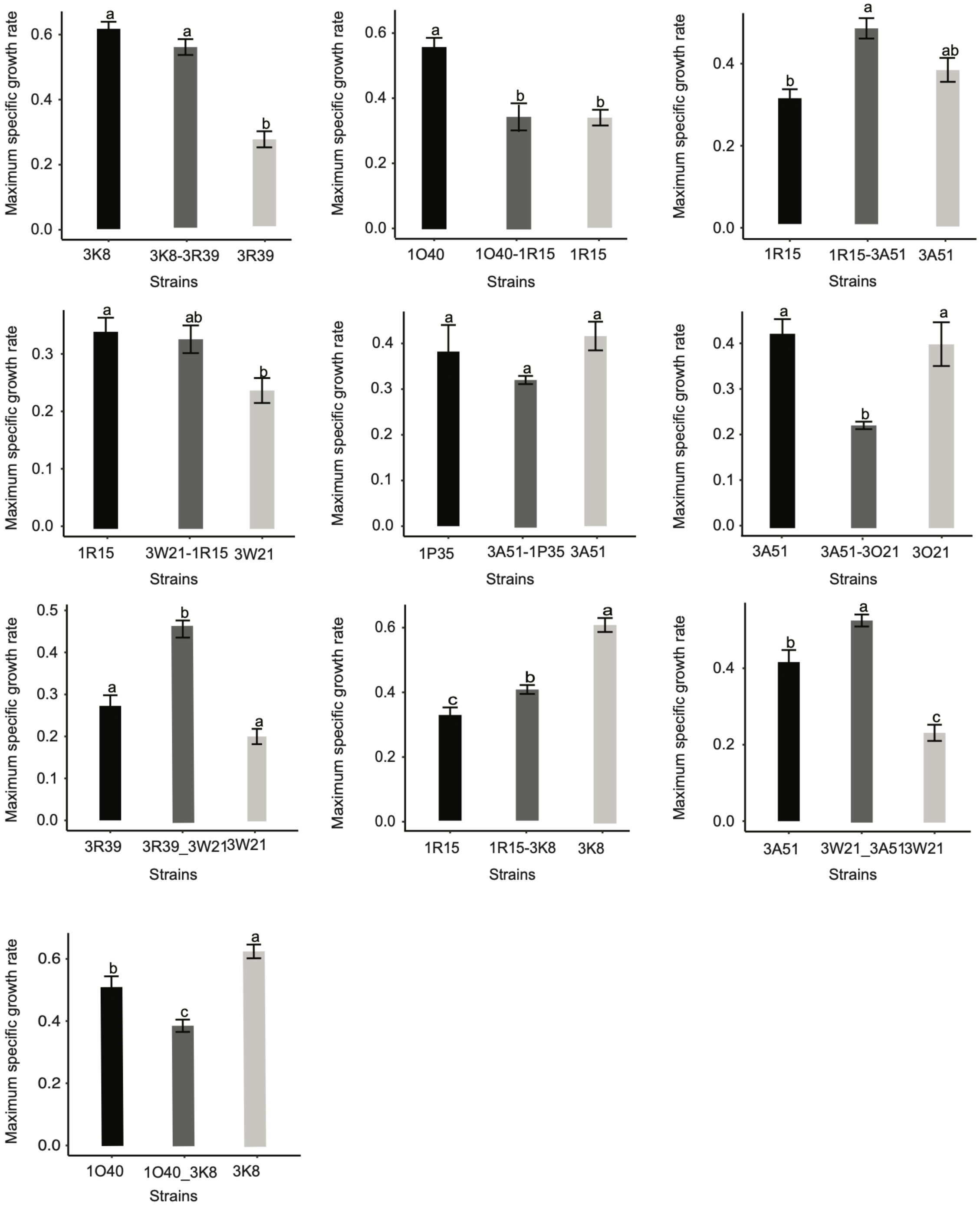

Supplementary Figure S3: Examples of pairwise strain interaction outcomes for *in vitro* cultures. The growth was assessed as the maximum specific growth rate (μmax) per day. Panels each

show single cultures (monocultures) as well as the corresponding mixed culture. Letters indicate significant differences using a Tukey-Kramer HSD test ($p > 0.05$).

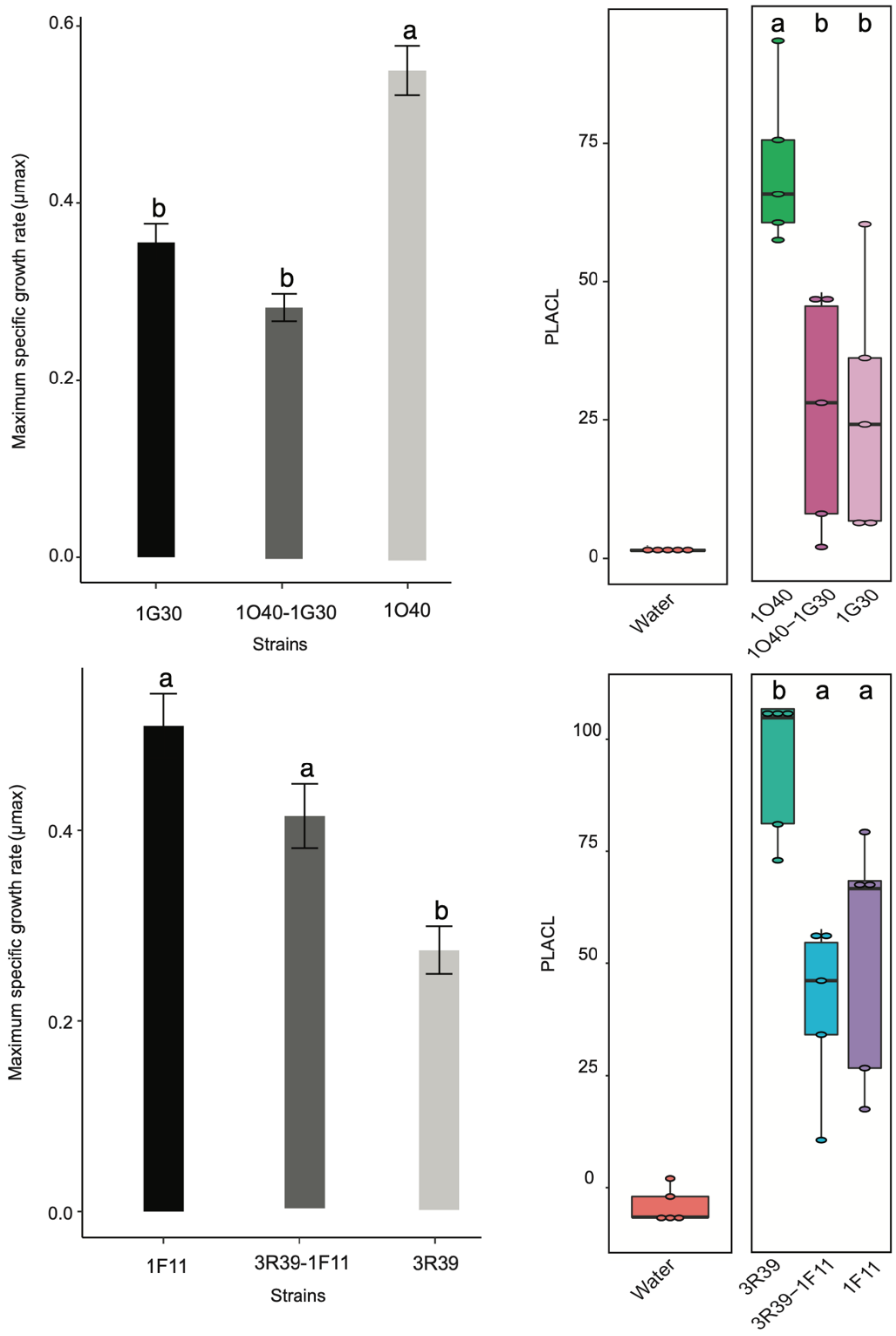

Supplementary Figure S4: Two examples of strain pairs with similar outcomes *in vitro* (left panels) and on the plant host (right panels). Panels each show single cultures (monocultures) as well as the corresponding mixed culture. Letters indicate significant differences using a Tukey-Kramer HSD test ($p < 0.05$).

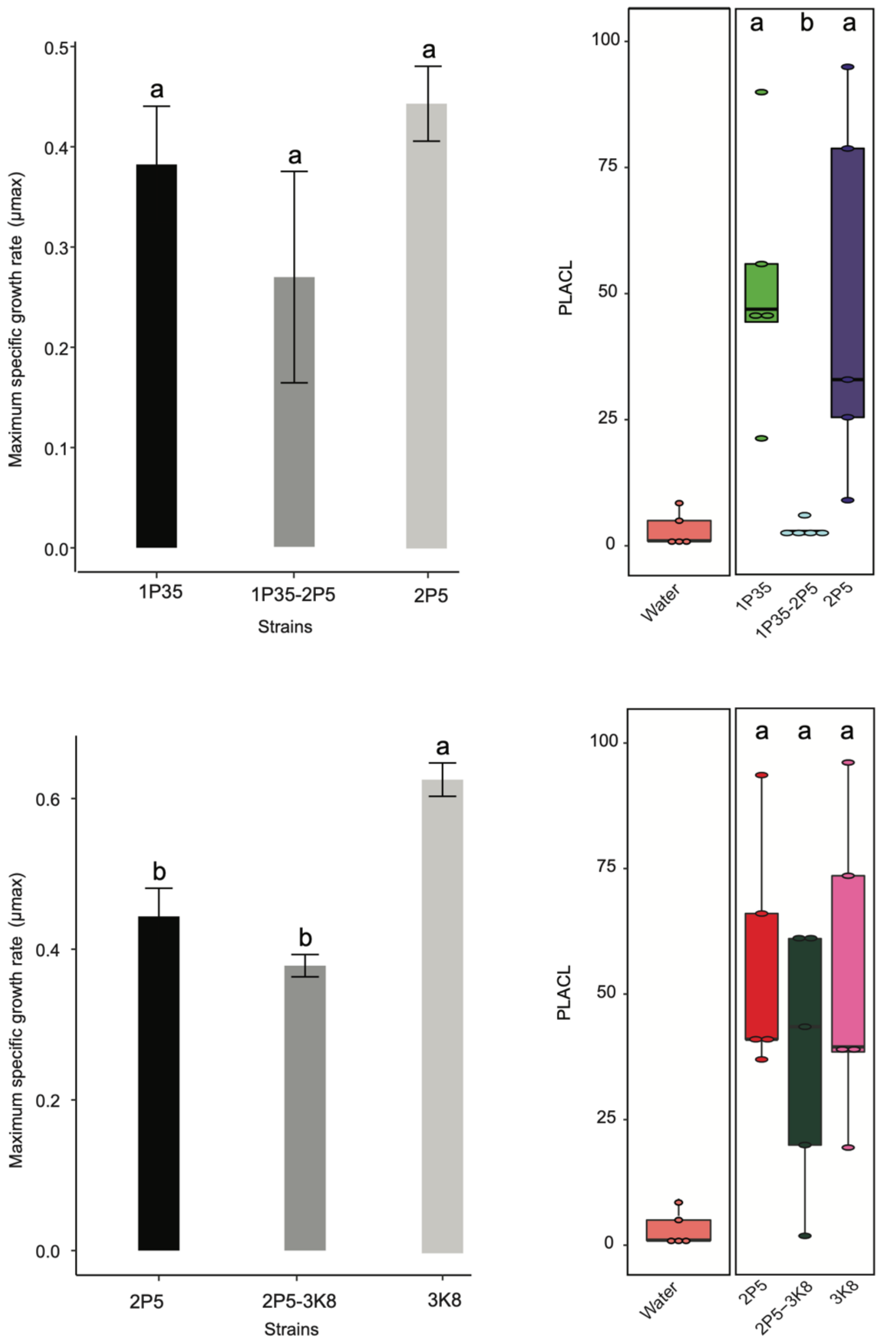

Supplementary Figure S5: Two examples of strain pairs with divergent outcomes *in vitro* (left panels) and on the plant host (right panels). Panels each show single cultures (monocultures) as well as the corresponding mixed culture. Letters indicate significant differences using a Tukey-Kramer HSD test ($p < 0.05$).
